# Rib Fractures in Trauma Patients: Prevalence, Associated Injuries, and Mortality Trends

**DOI:** 10.7759/cureus.70137

**Published:** 2024-09-24

**Authors:** Hüseyin Sami Yüksel, Mehmet Ali Aslaner, Secdegül Coşkun Yaş, Busegül Karakök, Ahmet Demircan

**Affiliations:** 1 Department of Emergency Medicine, Gazi University, Ankara, TUR; 2 Department of Emergency Medicine, Ankara Training and Research Hospital, Ankara, TUR; 3 Department of Emergency Medicine, Gümüşhane State Hospital, Gümüşhane, TUR

**Keywords:** abdominal injuries, emergency medicine, multiple trauma, rib fractures, thoracic injuries

## Abstract

Introduction

Rib fractures are a common consequence of thoracic trauma, often signaling the presence of additional injuries and increasing the risk of mortality. This study aimed to elucidate the frequency, patterns, and mortality rates associated with rib fractures in multitrauma patients and to explore the association between rib fractures and other systemic injuries.

Methods

In a retrospective observational study at a tertiary care hospital, electronic medical records of multi-trauma patients over a four-year period were examined. Inclusion criteria encompassed adult patients who had undergone whole-body or chest and abdomen CT imaging. The study evaluated the incidence and location of rib fractures, associated thoracic and intra-abdominal injuries, and the in-hospital mortality rate.

Results

Among the 1,338 patients analyzed, rib fractures were identified in 273 (20.4%), with a median age of 33 years and 199 (72.9%) were male. Motor vehicle accidents were the most common trauma mechanism with a rate of 52% (n=142). Patients with rib fractures had significantly higher occurrences of pneumothorax (25.3%), hemothorax (20.9%), and intra-abdominal injuries (17.5%) (p<0.001). Notably, patients with six or more rib fractures exhibited a higher frequency of liver, spleen, and kidney injuries (p=0.003, p=0.011, p=0.001, respectively). The in-hospital mortality rate was 5.9% (n=17) for patients with rib fractures, significantly higher than those without (p<0.001).

Conclusion

Rib fractures in multi-trauma patients are an important indicator of severe concurrent injuries and are associated with a higher mortality rate. These findings advocate for a systemic and immediate evaluation of patients with rib fractures upon presentation in emergency departments to improve survival outcomes.

## Introduction

Trauma accounts for 8% of global mortality, with thoracic trauma being a primary cause of death in about one-quarter of multiple-trauma patients and contributing to a mortality rate as high as 50% when combined with other injuries [1,2]. Rib fractures represent the most prevalent thoracic injury, occurring in up to 40% of cases [3].

Many patients with rib fractures are accompanied by additional injuries. In studies conducted on this patient group, isolated rib fracture was observed in only 6-12% of patients [4,5]. Other thoracic injuries such as pneumothorax, hemothorax, and pulmonary contusion are frequently observed in patients with rib fractures [3]. Additionally, there are studies on the relationship between intra-abdominal and other systemic injuries accompanying rib fractures [4,6]. Although it is rare for the fracture itself to be a life-threatening injury in patients with a rib fracture, the lives of these patients are usually threatened by other associated injuries. Prompt identification of rib fractures and any accompanying visceral injuries is crucial for assessing trauma severity, determining treatment strategies, and projecting patient outcomes.

The aim of this study was to ascertain the prevalence, number, and mortality of rib fractures in multi-trauma patients admitted to the emergency department, to determine the difference of pathologies detected in different systems between patients with and without rib fractures, and to examine the spectrum of thoracic, intra-abdominal, and other system injuries accompanying rib fractures, as well as their relationship with the location and level of the fractures.

## Materials and methods

The study was designed as a retrospective, observational analysis conducted at the Emergency Department of Gazi University Hospital, Ankara, Türkiye. Initiation of the study followed the acquisition of approval from the Gazi University Ethics Committee (approval number: E356084, dated 11.05.2022). The patient cohort consisted of multi-trauma individuals aged 18 and older who presented to the emergency department between 2018 and 2021 and underwent chest, abdomen, or whole-body computed tomography (CT) scans. Electronic medical records were reviewed to select patients. Exclusion criteria included multi-trauma patients without CT imaging of the chest and abdomen, individuals with penetrating trauma, those under the age of 18, and patients with non-traumatic rib fractures.

The electronic medical records of the patients included in the study were analyzed, and age, gender, mechanism of trauma, CT scans, localization of rib fracture, number of rib fractures, type and localization of thoracic and abdominal injuries, presence of intracranial, maxillofacial, pelvic, and vertebral injuries, and in-hospital mortality were recorded. The identified trauma mechanisms included motor vehicle accidents, pedestrian accidents, motorcycle accidents, falls, assaults, and train accidents. Rib fracture diagnoses were established based on CT findings, which were also assessed for other injuries by radiologists at the participating hospital.

Statistical analysis

The statistical analysis was performed using IBM SPSS Statistics for Windows, Version 25.0 (Released 2017; IBM Corp., Armonk, New York, United States). Frequency, mean, and standard deviation values were reported for the data. The normality of data distribution was evaluated using the Kolmogorov-Smirnov test. Categorical variables were statistically analyzed using Pearson’s chi-square and Fisher’s exact test. A p-value of less than 0.05 was considered statistically significant.

## Results

The data of a total of 1,338 trauma patients were analyzed. Whole-body CT scans were performed on 1,290 (96.4%) patients, while the remaining 48 (3.6%) underwent CT scans of only the thorax and abdomen. Of all patients, 73.2% were male, and the median age was 35 years. The most common mechanism of trauma was motor vehicle accidents with 669 (50%) patients. Rib fractures were detected in 273 (20.4%) of the analyzed patients. Of the patients with rib fractures, 72.9% were male and the median age was 33 years. Motor vehicle accidents were also the most frequent cause of rib fractures, affecting 142 (52%) patients (Table 1).

**Table 1:** Details of trauma patients presented to the emergency department

Characteristics	All trauma patients (n= 1338)	Patients with rib fractures (n= 273)
Male gender, n (%)	980 (73.2%)	199 (72.9%)
Age, median (min-max)	35 (18-94)	33 (18-88)
Trauma mechanism, n (%)		
Motor vehicle accidents	669 (49.9%)	142 (52%)
Motorcycle accidents	232 (17.3%)	48 (17.5%)
Fall	173 (12.9%)	34 (12.5%)
Pedestrian accidents	135 (10.1%)	30 (11%)
Assault	123 (9.1%)	19 (7%)
Train accident	6 (0.7%)	0 (0%)
In-hospital mortality, n (%)	21 (1.5%)	17 (5.9%)

The location of rib fractures was categorized as right, left, or bilateral, while the levels were classified into upper (1-4), median (5-8), lower (9-12), and multiple-level fractures. The majority of rib fractures were on the left side, involving 113 (41.4%) patients, and predominantly in the upper ribs, seen in 151 (55%) patients (Table 2).

**Table 2:** Location and level of rib fractures

	n (%)
Location of rib fracture	
Right	93 (34%)
Left	113 (41%)
Bilateral	67 (24%)
Level of rib fracture	
Upper (1-4)	151 (55%)
Median (5-8)	55 (20%)
Lower (9-12)	20 (8%)
Multiple levels	47 (17%)

Among the accompanying injuries in patients with rib fractures, the most common injuries were pneumothorax (25.2%) in the thorax and spleen injury (8.4%) in the abdomen. Some patients had multiple organ injuries.

The number of intra-thoracic injuries, hemothorax, pneumothorax, intra-abdominal injuries, spleen, liver, renal injuries, and intra-abdominal free fluid was significantly higher in patients with a rib fracture compared to those without a rib fracture (p<0.001). The prevalence of other accompanying injuries is delineated in Table 3.

**Table 3:** Comparison of patients with and without rib fracture * One patient could have more than one injury; ^+^ Chi-square test and Fisher’s exact test (for small sample sets)

Type of injury	Rib fracture		P^+^
	Absent (n= 1065), n (%)	Present (n= 273), n (%)	
Intraabdominal injury*	22 (2%)	48 (17.5%)	< 0.001
Spleen injury	9 (0.9%)	23 (8.4%)	< 0.001
Liver injury	4 (0.4%)	18 (6.5%)	< 0.001
Kidney injury	1 (0.1%)	11 (4%)	< 0.001
Intraabdominal free fluid	8 (0.7%)	9 (3.2%)	< 0.001
Intrathoracic injury*	125 (11.7%)	134 (49.2%)	< 0.001
Hemothorax	2 (0,2%)	57 (20.9%)	< 0.001
Pneumothorax	12 (1.1%)	69 (25.3%)	< 0.001
Contusion	34 (3.2%)	64 (23.4%)	< 0.001
Intracranial injury	82 (7.7%)	36 (13.2%)	0.004
Maxillofacial fracture	126 (11.8%)	55 (20.1%)	< 0.001
Vertebral fracture	155 (14.6%)	83 (30.4%)	< 0.001
Pelvic fracture	48 (4.5%)	44 (16.1%)	< 0.001
Scapular fracture	10 (1%)	31 (11.6%)	< 0.001
Clavicle fracture	22 (2.1%)	42 (15.4%)	< 0.001
Sternal fracture	14 (1.4%)	32 (11.8%)	< 0.001

Accompanying liver injury was found to be statistically significantly higher in right rib fractures (p=0.006). There was no significant relationship between the location of the rib fracture and splenic injury (p=0.61) (Table 4). Table 5 shows the relationship between the level of rib fracture and intra-abdominal injuries. Liver, spleen, and kidney injuries were found to be significantly higher in multiple-level rib fractures compared to others (p<0.001).

**Table 4:** Relation of rib fracture location and other injuries * Chi-square test

Other injuries	Rib fracture location			P*
	Right (n= 93), n (%)	Left (n=113), n (%)	Bilateral (n= 67), n (%)	
Liver injury	12 (13%)	2 (1.8%)	4 (6%)	0.006
Spleen injury	5 (5.4%)	12 (10.7%)	6 (9%)	0.61
Bilateral pneumothorax	4 (4.3%)	5 (4.4%)	10 (14.9%)	< 0.001
Left pneumothorax	0 (0%)	19 (16.8%)	8 (11.9%)	< 0.001
Right pneumothorax	14 (15.1%)	3 (2.7%)	6 (9%)	< 0.001
Bilateral hemothorax	1 (1.1%)	5 (4.4%)	7 (10.4%)	< 0.001
Left hemothorax	1 (1.1%)	18 (15.9)	9 (13.4%)	< 0.001
Right hemothorax	11 (11.8%)	1 (0.9%)	4 (6%)	< 0.001
Bilateral contusion	7 (7.5%)	8 (7.1%)	9 (13.4%)	< 0.001
Left contusion	1 (1.1%)	16 (14.2%)	5 (7.5%)	< 0.001
Right contusion	9 (9.7%)	2 (1.8%)	7 (10.4%)	< 0.001

**Table 5:** Relation of rib fracture level and intra-abdominal injuries *Chi-square test

Injuries	Level of rib fracture				P*
	Upper (1-4) (n= 151), n (%)	Median (5-8) (n=55), n (%)	Lower (9-12) (n= 20), n (%)	Multiple levels (n=47), n (%)	
Intra-abdominal injury	1 (0.7%)	6 (11%)	3 (15%)	38 (80.9%)	< 0.001
Liver injury	1 (0.7%)	3 (5.5%)	1 (5%)	13 (17.7%)	< 0.001
Spleen injury	0 (0%)	4 (7.3%)	2 (10%)	17 (36.2%)	< 0.001
Kidney injury	0 (0%)	0 (0%)	0 (0%)	11 (24.5%)	< 0.001

The relationship between the number of accompanying injuries and the number of rib fractures is shown in Table 6. Patients with six or more rib fractures exhibited a higher prevalence of liver, spleen, and kidney injuries (p=0.003, p=0.011, p=0.001, respectively).

**Table 6:** Association of number of rib fractures with intra-abdominal injuries and mortality *Chi-Square test

Accompanying injuries/mortality	Number of rib fracture			P*
	1 (n=64), n (%)	2-5 (n=146), n (%)	≥6 (n=63), n (%)	
Liver injury	2 (3.2%)	6 (4.2%)	10 (18.5%)	0.003
Spleen injury	0 (0%)	14 (9.6%)	9 (14.3%)	0.011
Kidney injury	0 (0%)	2 (1.4%)	9 (14.3%)	0.001
Mortality	0 (0%)	6 (4.1%)	9 (14.3%)	0.001

The in-hospital mortality rate in multi-trauma patients was 17 (5.9%) in patients with a rib fracture and four (0.4%) in patients without a rib fracture (p<0.001). Furthermore, mortality rates escalated in patients with six or more rib fractures compared to those with fewer fractures (p<0.001).

## Discussion

This study found that multi-trauma patients with rib fractures have a higher incidence of concomitant intra-thoracic and intra-abdominal injuries, as well as increased in-hospital mortality, compared to those without rib fractures. The most frequently occurring concurrent injuries in patients with rib fractures were intra-thoracic injuries and vertebral fractures, with pneumothorax and spleen injuries being the most common intra-thoracic and intra-abdominal injuries, respectively.

In our study of patients who underwent whole-body CT or chest and abdomen CT for multi-trauma, 20.4% were found to have rib fractures. In another study that analyzed CTs of multi-trauma patients admitted to the ED, the rate of rib fracture was 27% [7]. Motor vehicle accidents were the leading cause of multi-trauma, accounting for half of all cases. In patients with rib fractures, motor vehicle accidents were the most common cause of presentation, with a rate of 52%. This predominance is consistent with other studies, which also identify motor vehicle accidents as the primary cause of thoracic trauma and rib fractures. In the study by Okonta et al., 63% of patients with traumatic rib fractures were admitted due to motor vehicle accidents [8]. In the study by Sırmalı et al., motor vehicle accidents were the reason for presentation in 60.2% of patients with rib fractures [4].

The study observed that 49.2% of multi-trauma patients with rib fractures had intra-thoracic injuries, with pneumothorax occurring in 25.3% of cases. In the study by Karadayi et al., the most common accompanying chest injury in patients with rib fractures was pneumothorax, with a rate of 26.2% [9]. Parlak et al. found a pneumothorax rate of 36.7% in rib fracture patients [10]. Intra-abdominal injuries were associated with 17.5% of patients with rib fractures, and the most common injury was the spleen with 8.4%. Liver and spleen injuries, which are the two most common organ injuries in intra-abdominal injuries, had a similar percentage in different studies, although the ranking varied. Sharma et al. found 23% of concomitant abdominal solid organ injuries in patients with rib fractures [11]. The most common injury was splenic injury, with a rate of 9%. In Park's study, intra-abdominal injuries were associated with 21% of patients with rib fractures [3]. In this study, the most common injury was liver, followed by spleen injury. In the study by Parlak et al., 7.9% of patients had spleen injury, and 9.8% had liver injury [10].

Patients with six or more rib fractures showed a significantly higher rate of intra-abdominal organ injuries. This was an expected result since it was predicted that the energy of trauma in which more ribs were fractured would be higher. In the study by Kim et al., more intra-abdominal injuries were observed in patients with more than three rib fractures [12]. There are several studies in the literature reporting the association of lower rib fractures with intra-abdominal injury [4,13]. In our study, intra-abdominal injuries were more frequently observed in patients with rib fractures at multiple levels. In addition to the classic information that injuries to the lower ribs may be associated with intra-abdominal injury, the possibility of intra-abdominal injury should definitely be considered in patients with extensive chest trauma. Furthermore, rib fractures were significantly more frequent on the right side in patients with liver injury. Although left-sided fractures were more common in patients with spleen injury, this was not statistically significant.

In our study, the mortality rate was 5.9% in multi-trauma patients with rib fractures, 0.4% in those without, and 1.5% in all trauma patients. In another study, the mortality rate was 3% for all blunt trauma and 12% for patients with rib fractures [11]. Sirmali et al. found a mortality rate of 5.7% in patients with rib fractures [4], similar to our study. Brasel et al. found a mortality rate of 4% in patients with rib fractures [14]. Flagel et al. analyzed 67,221 patients with rib fractures and found that the mortality rate was significantly higher in patients with six or more rib fractures compared to patients with fewer rib fractures [15]. Similarly, in our study, the mortality rate was higher in patients with six or more rib fractures compared with the other group.

Moreover, in the current study, pneumothorax, hemothorax, intra-abdominal injuries including liver, spleen, and kidney, intracranial injuries, maxillofacial fractures, vertebral fractures, and pelvic fractures were more common in multi-trauma patients with rib fractures than in multi-trauma patients without rib fractures. In another study of all trauma patients, intracranial injuries, hemopneumothorax, solid organ injuries including liver, spleen, and kidney, and pelvic fractures were statistically significantly more common in patients with rib fractures than in patients without rib fractures [11]. This highlights the importance of a thorough evaluation of additional systemic pathologies in multi-trauma patients with rib fractures.

The single-centre and retrospective design of the study were the main limitations. Admissions and diagnoses of the patients were analyzed only through electronic medical records, and hence, data of patients with missed diagnoses and patients diagnosed only by X-ray could not be included in the study. The fact that the threshold for CT scanning in trauma patients is perceived differently by physicians and that there are no clear limits on this issue may have created heterogeneity in the severity of trauma in the included patients. We may have missed multi-trauma patients who did not have chest and abdominal trauma together. In addition, we did not evaluate whether patients had displaced rib fractures. As previous studies have shown, this may cause associated injuries [10].

## Conclusions

Rib fractures in multi-trauma patients are a critical indicator of concurrent severe injuries and are associated with a higher mortality rate, especially when six or more ribs are involved. Motor vehicle accidents emerge as the most common cause, with pneumothorax and splenic injuries as frequent companions. These findings underscore the urgent need for comprehensive evaluation and management of rib fractures in trauma care to improve patient prognosis.
